# Surgical and demographic trends in genital gender-affirming surgery in transgender women: 40 years of experience in Amsterdam

**DOI:** 10.1093/bjs/znab213

**Published:** 2021-07-19

**Authors:** W B van der Sluis, I de Nie, T D Steensma, N M van Mello, B I Lissenberg-Witte, M -B Bouman

**Affiliations:** Department of Plastic, Reconstructive and Hand Surgery, Amsterdam UMC, location VUMC, Amsterdam, The Netherlands; Centre of Expertise on Gender Dysphoria, Amsterdam UMC, location VUMC, Amsterdam, The Netherlands; Centre of Expertise on Gender Dysphoria, Amsterdam UMC, location VUMC, Amsterdam, The Netherlands; Department of Endocrinology, Amsterdam UMC, location VUMC, Amsterdam, The Netherlands; Centre of Expertise on Gender Dysphoria, Amsterdam UMC, location VUMC, Amsterdam, The Netherlands; Department of Medical Psychology, Amsterdam UMC, location VUMC, Amsterdam, The Netherlands; Centre of Expertise on Gender Dysphoria, Amsterdam UMC, location VUMC, Amsterdam, The Netherlands; Department of Gynaecology and Obstetrics, Amsterdam UMC, location VUMC, Amsterdam, The Netherlands; Department of Epidemiology and Data Science, Amsterdam UMC, location VUMC, Amsterdam, The Netherlands; Department of Plastic, Reconstructive and Hand Surgery, Amsterdam UMC, location VUMC, Amsterdam, The Netherlands; Centre of Expertise on Gender Dysphoria, Amsterdam UMC, location VUMC, Amsterdam, The Netherlands

## Abstract

This was a single-centre, retrospective study of transgender women undergoing genital gender-affirming surgery. A chart study was conducted, recording individual demographics, all genital surgical procedures, and surgical techniques. Procedure incidence, techniques employed, and demographic variations over the years were analysed.

## Introduction

The number of transgender people seeking medical and surgical care is increasing worldwide^1^^,^^2^. Genital gender-affirming surgery (gGAS) in transgender women may comprise bilateral orchiectomy, vaginoplasty, or gender-confirming vulvoplasty (GCV). Vaginoplasty combines penectomy, orchiectomy, labiaplasty, clitoroplasty, and creation, and lining, of a neovaginal canal. In GCV, no neovaginal canal is dissected and only external female genitalia are constructed.

Penile inversion vaginoplasty is the vaginoplasty standard, in which a penile skin flap is used for the neovaginal lining^3^. Alternatives are full- or partial-thickness skin graft, or intestinal or peritoneal vaginoplasty^4–6^. Genital anatomy may influence the chosen technique. This article provides information on demographic and surgical gGAS trends among transgender women in the authors’ institution.

## Methods

The majority of surgical transgender healthcare countrywide is performed in the authors’ centre, making it suited for analyses of demographic and surgical trends. In the diagnostic and treatment phases, the World Professional Association for Transgender Health Standard of Care is followed^7^. Individuals may opt for gGAS after thorough psychological screening, 12 months of hormone treatment, more than 6 months of testosterone suppression, surgical eligibility screening, and multidisciplinary consultation. In this centre, smoking, BMI below 18 and above 30 kg/m^2^ are considered contraindications to vaginoplasty and GCV. Institutional review board approval of the study protocol was obtained (METC2014322).

### Retrospective chart study

All transgender women who underwent primary gGAS between January 1980 and January 2020 were identified from a departmental database.

A retrospective chart study was performed, with recording of gGAS procedures, surgical (sub)techniques, individual demographics (age, previous use of puberty-suppressing hormones, age, and Tanner stage at start of puberty suppression, BMI, history of smoking and drug abuse, fertility preservation), neovaginal depth, measured after surgery and, if present, revision vaginoplasty procedures, techniques, and indications.

Sexual orientation (towards men, towards women, towards both men and women, asexual, unclear for the individual) has been asked at surgical intake at the outpatient clinic since 2011 as an exploratory step in the assessment of postoperative sexual desires.

Procedure incidence, techniques employed, and demographic variations over the years were analysed.

### Statistical analysis

Categorical variables were compared using the χ^2^ test; independent-samples *t* test or ANOVA was used for normally distributed continuous variables, and the Mann–Whitney *U* test or Kruskal–Wallis test for those with a non-normal distribution. Predictors for choosing fertility preservation were identified using backward logistic regression.

## Results

### Trends in genital gender-affirming surgery

A total of 1531 transgender women underwent gGAS at this institution during the study period. The number of transgender women undergoing gGAS increased over time, particularly in recent years (*Fig. S1*).

**Fig. 1:**
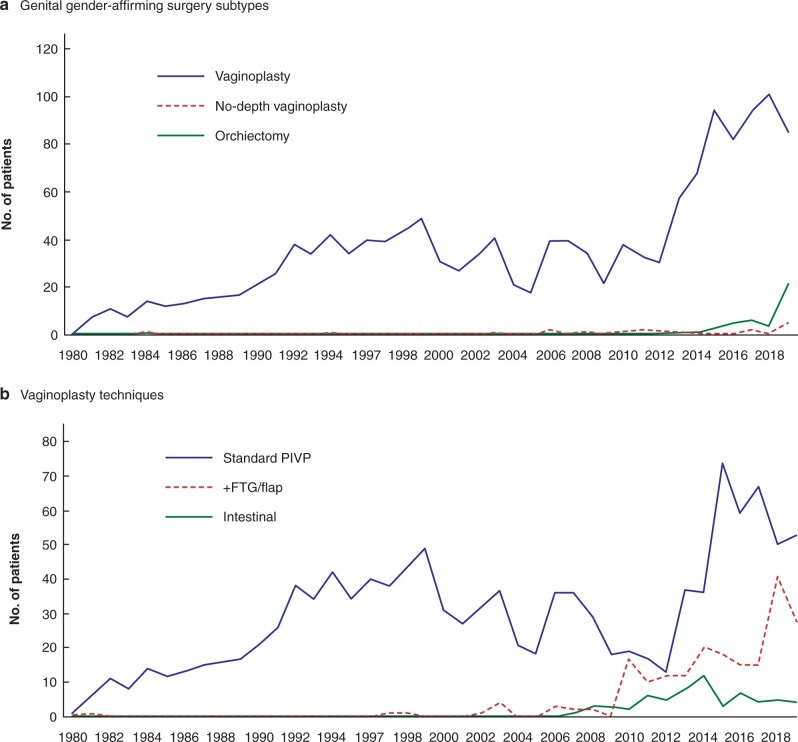
Genital gender-affirming surgery subtypes and vaginoplasty techniques over time. **a** Genital gender-affirming surgery subtypes and **b** vaginoplasty techniques. PIVP, penile inversion vaginoplasty; FTG, full-thickness skin graft.

Of the 1531 transgender women, 1468 (95.9 per cent)underwent vaginoplasty (1405 penile inversion, 63 intestinal vaginoplasty), 19 (1.2 per cent) GCV, and 44 (2.9 per cent) orchiectomy (*Table 1*). Mean(s.d.) neovaginal depth after vaginoplasty was 13.1(2.1) cm. Before 2002, almost all transgender women underwent vaginoplasty as gGAS. More recently, orchiectomy and GCV were requested more frequently. For example, in 2019, 85 vaginoplasty procedures (77 per cent), 21 orchiectomies (19 per cent), and five GCVs (5 per cent) were performed (*Fig. 1*)^8^^,^^9^.

**Table 1:** Demographics of transgender women undergoing primary genital gender-affirming surgery at the authors’ institution between January 1980 and January 2020

	Total	Vaginoplasty	Orchiectomy	GCV	***P***
	(*n* = 1531)	(*n* = 1468)	(*n *= 44)	(*n* = 19)	
Age at surgery (years)*	33 (25–44)	33 (24–44)	32 (26–45)	54 (45–60)	<0.001^#^
**BMI at surgery (kg/m^2^)** ^†^	23.7(3.5)	23.6(3.3)	26.4(6.6)	24.0(2.4)	<0.001**
**History of puberty suppression**	135 (8.8)	132 (9.0)	3 (6.8)	0 (0)	0.355
**Sexual orientation at surgical intake** ^‡^	699 (100)	645 (100)	42 (100)	12 (100)	<0.001
Solely towards men	372 (53)	357 (55)	13 (31)	2 (17)	
Solely towards women	217 (31)	194 (31)	15 (36)	8 (75)	
Towards both men and women	85 (12)	75 (12)	9 (21)	1 (8)	
Asexual	4 (1)	1 (0.2)	2 (5)	1 (8)	
Unclear for the individual	14 (2)	11 (2)	3 (7)	0 (0)	
Unknown to researcher	7 (1)	7 (1)	0 (0)	0 (0)	
**Opted for fertility preservation** ^§^	117 of 1047 (11)	109 of 987 (11)	6 of 43 (14)	2 of 17 (12)	0.832

‡ Values in parentheses are percentages unless indicated otherwise; values are median (i.q.r.) and ^†^mean (s.d.). Data available for 2011–2019; ^§^data available for 2000–2019. GCV, gender-confirming vulvoplasty. ^¶^χ^2^ test, except ^#^Kruskal–Wallis test and **ANOVA.

Intestinal vaginoplasty was performed as the primary gGAS procedure since 2007 and undertaken 63 times since. Surgical indications were: penoscrotal hypoplasia owing to a history of puberty suppression (46 procedures), shortage of penile skin due to circumcision (7), and biological variation (10). A sigmoid (62) or ileal (1) segment was used as neovaginal lining.

Of 1468 primary vaginoplasty procedures, 34 individuals (2.3 per cent) underwent revision vaginoplasty. Indications and techniques for revision vaginoplasty are provided in *Table S1*. Currently, laparoscopic intestinal vaginoplasty is preferred for this indication, because of the lower risk of rectal perforation^10^.

Median age at the time of surgery was 33 (i.q.r. 25–44) years (*Figs S2* and *S3*). Individuals who opted for GCV were generally older, had no history of puberty suppression, and were more frequently sexually oriented towards women (*Table 1*).

### Puberty suppression

An increase in individuals with a history of puberty suppression was observed, from 2000 (2 of 31) to 2019 (18 of 111, 16 per cent) (*Fig. S4*). Age and Tanner stage at the start of puberty suppression influenced the vaginoplasty technique chosen. When puberty suppression was started at Tanner stage G2–3, penoscrotal hypoplasia led to the choice of intestinal vaginoplasty in approximately 70 per cent of individuals (*Table S2*).

### Fertility preservation

Over time, more transgender women opted for semen cryopreservation (*Fig. S5*). In 2000, 1 of 31 transgender women opted for semen cryopreservation before gGAS. In 2019, this number increased to 28 of 111 (25 per cent). Being younger at time of gGAS (odds ratio (OR) 0.90, 95 per cent c.i. 0.87 to 0.93; *P* < 0.001), having a history of puberty suppression (OR 5.01, 2.42 to 10.41; *P* < 0.001), and undergoing gGAS more recently (OR 1.21, 1.12 to 1.34) were identified as predictors for choosing for fertility preservation.

## Discussion

Parallel to the observed increase in transgender women seeking psychological and medical care, a drastic increase in gGAS procedures was observed. Vaginoplasty is the most commonly performed gGAS procedure, although a rise in orchiectomy procedures was observed in 2019.

The observed surgical trends are a result of patient-related factors (more transgender individuals seeking medical care), societal factors (changed laws, increased awareness and recognition of gender diversity, insurance policies, technological factors), and institutional factors (capacity, availability of specific surgical skills). Currently, the choice of a specific gGAS subtype is based on a combination of surgical and anatomical possibilities, and individual preferences. GCV and orchiectomy as surgical options may be a result of client-centred care and shared-decision making.

Individuals undergoing specific subtypes of gGAS represent unique subgroups, with intergroup demographic differences. Reported sexual orientation was more frequently towards men in the vaginoplasty group, which may reflect postoperative sexual desires. Individuals undergoing GCV were older and did not have a history of puberty suppression.

Puberty suppression has a positive effect on quality of life in transgender women who apply for treatment during their adolescent years^11^^,^^12^. An increase was observed in transgender women who used puberty-suppressing hormones opting for gGAS. When starting puberty suppression at a prepubertal or early pubertal stage (Tanner G2–3), penoscrotal hypoplasia may limit the surgical possibilities for vaginoplasty owing to a shortage of penoscrotal skin, which makes standard penile inversion vaginoplasty impossible. Alternative strategies, such as additional (scrotal) skin grafts/scrotal flaps or skin graft, intestinal or peritoneal vaginoplasty may be necessary^6^^,^^13^^,^^14^. This should be a point of attention for users and prescribers of puberty-suppressing hormones, and should be discussed with adolescents opting for this treatment^15^. If the percentage increase in individuals taking puberty-suppressing hormones continues, the incidence of non-standard vaginoplasty procedures will increase simultaneously. With increased use of puberty-suppressing hormones, gender surgeons may need to gain experience in alternative vaginoplasty techniques.

The number of transgender women opting for fertility preservation before gGAS increased over the years to 25 per cent in 2019, whereas rates reported in the literature vary from 0 to 62 per cent^16–19^. Since 2015, extensive fertility preservation counselling has been implemented in the authors’ clinic and semen cryopreservation costs have been covered by insurance. Improved availability of, and access to, fertility care may play a major role in its increased use. The increase in individuals starting puberty suppression at early pubertal stages, when serum testosterone concentrations are insufficient for spermatogenesis, may lead to an increase in individuals without options for preservation of fertility.

A strength of this study is the unique large study population in a centre with long-term gGAS experience. A limitation is its retrospective nature.

This study has identified remarkable demographic trends. The topic of fertility preservation and the influence of puberty suppression on surgical technique must be taken into account during counselling of transgender women.

*Disclosure*. The authors declare no conflict of interest.

## Supplementary material

Supplementary material is available at *BJS* online.

## Supplementary Material

znab213_Supplementary_Data
