# Supplementary material for: Surgical and demographic trends in genital gender-affirming surgery in transgender women: 40 years of experience in Amsterdam
Source: Br J Surg. 2021 Jul 19;109(1):8–11. doi: 10.1093/bjs/znab213 (PMC10364763; doi:10.1093/bjs/znab213)
Supplement: znab213_Supplementary_Data [file znab213_supplementary_data.zip › Supplementary_Table_1.docx]

**Supplementary Table 1. Indications and techniques for revision vaginoplasty**

| Indiation for revision vaginoplasty, n (%)  Total or partial neovaginal obliteration  Necrosis of the inverted penile skin flap  Insufficient neovaginal depth immediately after vaginoplasty  Severe neovaginal hypergranulation, refractory to conservative treatment  Neovaginal prolapse  Non-take and necrosis of used skin grafts | 23 (68%)  4 (12%)  2 (6%)  2 (6%)  2 (6%)  1 (3%) |
| --- | --- |
| Revision vaginoplasty technique, n (%)  Intestinal vaginoplasty  Full-thickness skin graft vaginoplasty  Abdominal grafts  Groin grafts  Slit-thickness skin graft vaginoplasty  Pudendal thigh flaps  Peritoneal vaginoplasty | 19 (56%)  10 (29%)  8 (24%)  2 (6%)  1 (3%)  3 (9%)  1 (3%) |
