# Supplementary material for: Surgical and demographic trends in genital gender-affirming surgery in transgender women: 40 years of experience in Amsterdam
Source: Br J Surg. 2021 Jul 19;109(1):8–11. doi: 10.1093/bjs/znab213 (PMC10364763; doi:10.1093/bjs/znab213)
Supplement: znab213_Supplementary_Data [file znab213_supplementary_data.zip › Supplementary_Table_2.docx]

**Supplementary Table 2. Vaginoplasty subtechnique in transgender women with a history of puberty suppression opting for vaginoplasty between 2000 and present.**

|  | Total  (n=128) | PIVP  (n=65) | PIVP + FTG/flap  (n=17) | Intestinal vaginoplasty  (n=46) | P-value |
| --- | --- | --- | --- | --- | --- |
| Mean age at start puberty suppression, y ± SD | 14.8 ± 1.8 | 15.9 ± 1.6 | 14.7 ± 1.8 | 13.5 ± 1.2 | P<0.01* |
| Mean penile length at surgery, cm ± SD | 11 ± 3.5 | 14.2± 1.7 | 11.1 ± 1.6 | 7.3 ± 1.3 | P<0.01* |
| Tanner stadium at start puberty suppression, n (%)   - G1 - G2 - G3 - G4 - G5 | -  24  25  11  68 | -  3 (13%)  3 (12%)  7 (64%)  52 (76%) | -  4 (17%)  4 (16%)  2 (18%)  7 (10%) | -  17 (71%)  18 (72%)  2 (18%)  9 (13%) | P<0.01** |
| Mean neovaginal depth after surgery, cm ± SD | 14.2 ± 1.7 | 13.5 ± 1.3 | 13.3± 1.8 | 16.1 ± 1.1 | P<0.01* |

PIVP Penile inversion vaginoplasty, FTG Full thickness skin graft, SD standard deviation

One-way ANOVA*, Chi-square**
