# Supplementary figures and images for: Surgical and demographic trends in genital gender-affirming surgery in transgender women: 40 years of experience in Amsterdam
Source: Br J Surg. 2021 Jul 19;109(1):8–11. doi: 10.1093/bjs/znab213 (PMC10364763; doi:10.1093/bjs/znab213)

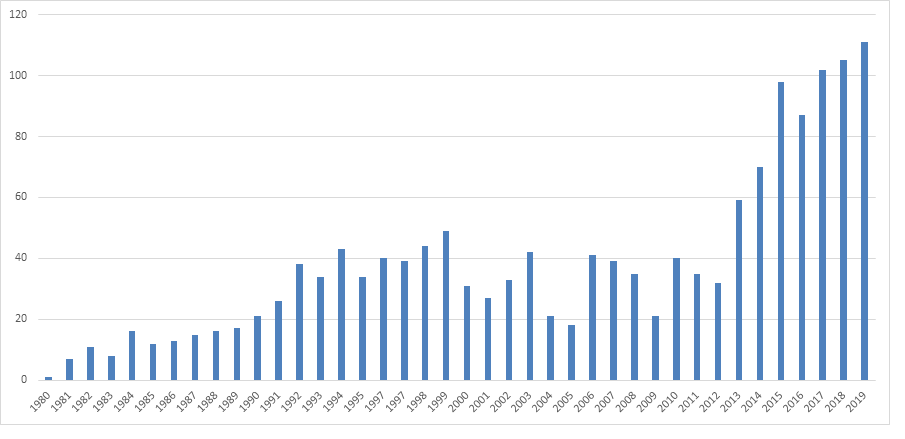

Supplement: znab213_Supplementary_Data [file znab213_supplementary_data.zip › Supplementary_Figure_1.tif]

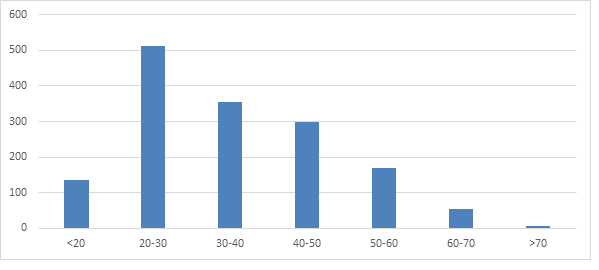

Supplement: znab213_Supplementary_Data [file znab213_supplementary_data.zip › Supplementary_Figure_2.tif]

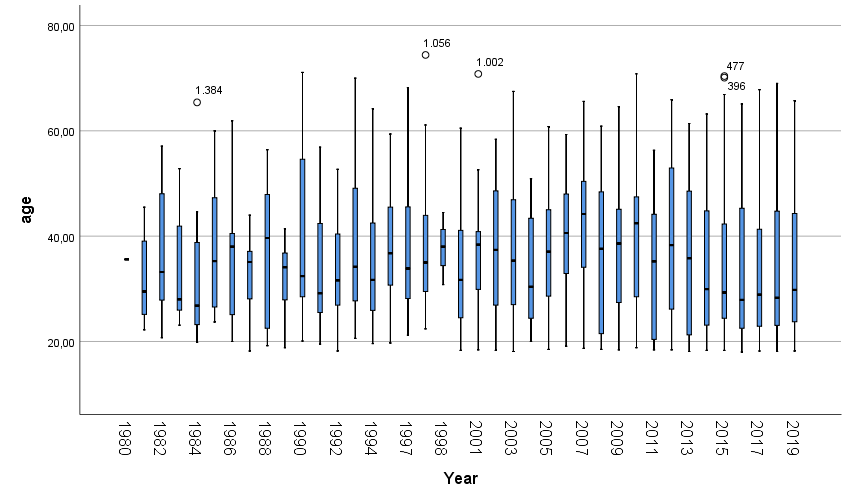

Supplement: znab213_Supplementary_Data [file znab213_supplementary_data.zip › Supplementary_Figure_3.tif]

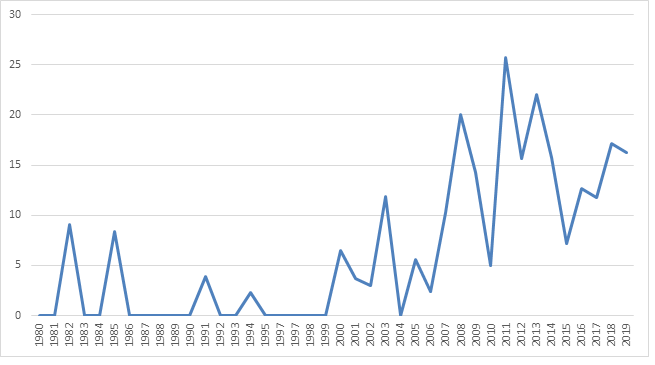

Supplement: znab213_Supplementary_Data [file znab213_supplementary_data.zip › Supplementary_Figure_4.tif]

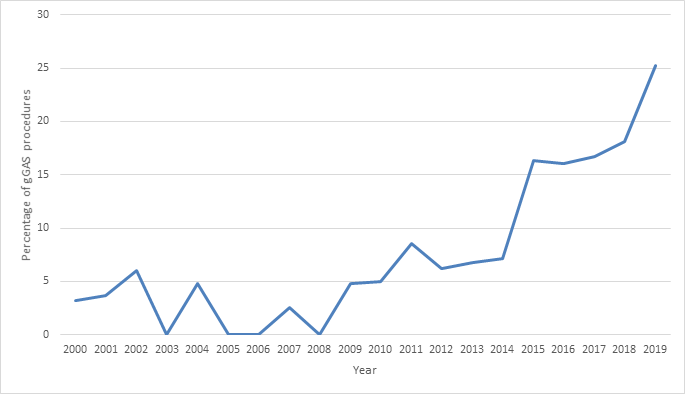

Supplement: znab213_Supplementary_Data [file znab213_supplementary_data.zip › Supplementary_Figure_5.tif]
